# Observation of a Large Landslide on La Reunion Island Using Differential Sar Interferometry (JERS and Radarsat) and Correlation of Optical (Spot5 and Aerial) Images

**DOI:** 10.3390/s90100616

**Published:** 2009-01-21

**Authors:** Christophe Delacourt, Daniel Raucoules, Stéphane Le Mouélic, Claudie Carnec, Denis Feurer, Pascal Allemand, Marc Cruchet

**Affiliations:** 1 Université Européenne de Bretagne, Université de Brest; CNRS; UMR 6538 Domaines Océaniques; Institut Universitaire Européen de la Mer, Place Copernic, 29280 Plouzané, France; 2 BRGM, Aménagement et Risque Naturels, 3 avenue Claude Guillemin, 45060 Orléans cedex 2, France; 3 Laboratoire de Planétologie et Géodynamique, CNRS/UMR 6112, Université de Nantes, 2 rue de la Houssinière, 44322 Nantes cedex, France; 4 Laboratoire Sciences de la Terre – UCBL&ENS-UMR 5570 CNRS Bat Géode 2 Rue Dubois 69622 Villeurbanne Cedex, France

**Keywords:** DInSAR, Optical Correlation, Landslide

## Abstract

Slope instabilities are one of the most important geo-hazards in terms of socio-economic costs. The island of La Réunion (Indian Ocean) is affected by constant slope movements and huge landslides due to a combination of rough topography, wet tropical climate and its specific geological context. We show that remote sensing techniques (Differential SAR Interferometry and correlation of optical images) provide complementary means to characterize landslides on a regional scale. The vegetation cover generally hampers the analysis of C–band interferograms. We used JERS-1 images to show that the L-band can be used to overcome the loss of coherence observed in Radarsat C-band interferograms. Image correlation was applied to optical airborne and SPOT 5 sensors images. The two techniques were applied to a landslide near the town of Hellbourg in order to assess their performance for detecting and quantifying the ground motion associated to this landslide. They allowed the mapping of the unstable areas. Ground displacement of about 0.5 m yr^-1^ was measured.

## 1. Introduction

Landslides are a common disturbance in tropical mountainous areas [1–3]. Due to specific geological and climatic conditions, landslides are a major hazard affecting the island of La Réunion in the Indian Ocean [4, 5]. Several large landslides (up to several square kilometres) severely affect infrastructures (especially roads) and habitations mostly in the region of the “Circles”. The Hellbourg landslide in the Salazie Circle has been monitored by GPS for several years on regularly revisited points. It represents a serious hazard for inhabited sectors and it could have an impact on buildings, roads and population. The speed of the landslide has a nearly linear evolution with time according to the GPS measurements performed during the period 1997–2000 by the Regional Geological Survey of la Réunion Island [6]. The averaged displacement rate is 0.5 m·y^-1^.

Presently, most of the techniques for monitoring landslide displacements are derived from measurements from ground based reference stations. Conventional geodetic (triangulation, tacheometry) and extensometry techniques are the most widely used [7], along with GPS surveys [8–10]. The data acquired using these techniques are available only for major landslides and are limited to the last 15 (GPS) or 20 (laser) years. Moreover, due to the spatial and temporal heterogeneities of ground displacements, such ground-based measurements are often not accurate enough to fully describe the velocity field of a landslide. These techniques also require additional human intervention on the landslide or in its vicinity.

On the other hand, remote sensing imagery provides a powerful tool to measure landslide displacements because it offers a synoptic view of the landslide that can be repeated at different time intervals. Furthermore, this technique is efficient at various scales (from the individual landslide to regional scale observation). Remote sensing techniques such as Differential SAR interferometry (DInSAR) and optical image correlation, which are based on an existing image archive, provide a way to investigate, possibly since their onset, ground displacement events.

DInSAR has been widely used for the detection of surface movements associated with various geophysical phenomena. Previous studies on landslides have been carried out in the C-band [11–13] and the L-Band [14] in an Alpine context. These studies have already showed the potential, for specific test cases, of DInSAR to give a synoptic view of the displacement.

One other remote sensing technique based on correlation of optical images can be applied in order to obtain 2D horizontal displacement maps (field of displacement occurred between the two acquisitions projected in the horizontal plan). This methodology has been applied to aerial and satellite images to measure the displacements generated by various geophysical phenomena. Few studies have also been made over landslides [15–19]. In this paper, we investigate the Hellbourg landslide displacements in La Réunion Island by integrating both JERS-1 and RADARSAT DInSAR with the results of the correlation of optical aerial and SPOT 5 images.

## 2. Differential Radar Interferometry (DInSAR)

### 2.1. Principle

DInSAR provides information on the change in distance through time between the ground and the satellite sensor. The technique is based on the change in phase between two Synthetic Aperture Radar (SAR) acquisitions. Details on the principles of radar differential interferometry can be found in [20, 21]. A differential interferogram is a map of the surface displacement along the line of sight occurring between the acquisition dates of the two images. The DInSAR technique has been successfully applied for detecting and mapping surface displacements caused by natural and anthropic phenomena such as earthquakes [22], ice stream flows [23], volcanic activity [24] and land subsidence [25, 26].

However, some severe limitations prevent systematic use of this technique. The major limitation is the loss of signal coherence [20]. This loss of coherence is due to changes in the physical and geometric characteristics of the targets. The signal is also affected by atmospheric effects [27], related to fluctuations of the atmosphere between the two image acquisitions, changing the refractive index of the layer crossed by the wave, and therefore, its optical path (atmospheric delay). They can have similar signatures as ground displacement [28]. The loss of coherence produces a high spatial frequency phase noise, whereas atmospheric effects produce lower spatial frequencies that can be misinterpreted as displacements [29]. In our study, these limitations were enhanced by specificities of the local context. The dense vegetation that covers most of La Reunion is responsible for low coherence in the C-band. In the study area, the tropical climate and the steep, high relief (up to 3,000 m) increases the possibility of fluctuations of the atmospheric layer over the studied area. As shown by [14, 30], the L-band of radar sensors (wavelength ranges between 21.41 and 24.57 cm) generally provides a higher coherence than the C-band (wavelength ranges between 5.06 to 5.71 cm) over non-urban areas. Indeed, due to its higher wavelength, L-band waves penetrate deeper into the vegetation cover and provide information from the more stable scatterers located on the ground surface. It is, therefore, less sensitive to temporal decorrelation due to vegetation changes between two acquisitions. As pointed out by [31], the DInSAR technique is well adapted for the detection and monitoring of landslides on the slopes facing away from the SAR look vector. In addition, the ground displacement rates have to be compatible with the time sampling of the DInSAR technique. The L-band is particularly adapted to investigate decimetric displacements In this case, one interferometric fringe corresponds to a movement of 11.25 cm (half of the wavelength) in the line of sight of the satellite (corresponding to 2.8 cm for the C-band). Therefore, time spans of several months are required to detect displacement rates of few centimetres per month with the L band. Displacements rates larger than decimetres per month cannot be detected by L-band DInSAR (and C-band DInSAR is similarly limited to ∼1.5 cm per month).

### 2.2. Data set

The ERS (European Remote Sensing Satellite) archive is the most widely used data set for interferometric applications. However, no ERS image has been acquired over La Réunion because of the lack of an onboard data recorder and a neighbouring reception station. We have therefore used a set of 14 Radarsat Single Look Complex images (C-band, S3 mode, spatial sampling of 5.1 m in azimuth × 11.6 m in range) acquired between the January 26^th^ 1999 and January 10^th^ 2002. In addition, six JERS-1 level 0 images (L-band, spatial sampling of 8.9 m in azimuth × 8.8 m in range) covering the period of January 1997 to August 1997 have been processed. No other L-band InSAR data is available for the island. We used the Gamma interferometric software [32] – for details on the processing, the reader can refer to the Gamma system description (http://www.gamma-rs.ch/no_cache/software/system-overview.html) - complemented by procedures developed in IDL language [33]. An adaptive filter [23] with a window size of 32 pixels and a coefficient of 0.7 has been applied to improve the signal-to-noise ratio of the interferograms. In the procedure proposed by [23], this coefficient is used as a power of the amplitude of the Fourier transform of the complex data values estimated on the filtering window. The result is a band-pass filtering adapted to the phase gradient. These parameters provided the best trade-off between fringe smoothing and detection capabilities.

All the interferometric combinations have been automatically produced from the 14 Radarsat scenes. Table 1 shows the characteristics of the eleven most interesting pairs in terms of baseline and temporal coverage. Table 2 summarizes the characteristics of the JERS-1 interferograms that have been similarly produced. For the correction of the phase topographic component in the interferograms, we used a DEM produced by IGN (Institut Géographique National) with a 25 m resolution.

## 4. Results

**Figure 1.**
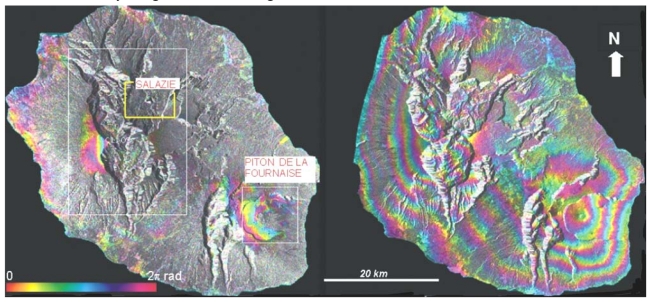
Left: one of the most coherent Radarsat C-band interferograms (15/05/2001 – 23/11/2001, perpendicular baseline=68 m). Right: one of the most coherent JERS-1 (02/01/1997-14/05/1997, perpendicular baseline=187 m) interferograms obtained for the whole island. The white rectangles on Figure 1 indicate the Circles sector and the Piton de la Fournaise volcano. The yellow rectangle shows the area displayed in Figure 2. No coherence loss is observed in the L-band despite the dense vegetal cover of the island. The SAR intensity image is used as background.

On a regional scale, a good coherence level of the Radarsat interferograms is observed for bare soils and urbanized areas (Figure 1, left). Furthermore, significant fringe patterns are observed on the Piton de la Fournaise volcano. This corresponds to displacements of the volcanic edifice due to the injection of magma into dykes during eruptive events, which took place between 1999 and 2002.

A similar observation was made by [34] for a previous eruption. This volcanic displacement will be not discussed in this paper since our focus is on landslides. Unfortunately, a low coherence level was systematically obtained with Radarsat data in all areas covered by vegetation which are the key areas for the landslides issue. This prevents the use of most of the C-band DinSAR interferograms. On the other hand, the JERS-1 interferograms are coherent on the whole island, even for time spans of several months (Figure 1, right). The L-band penetrates deeper into the vegetation cover. A comparison of the capabilities of the two sensors for exactly the same period of time was not possible due to the very sparse image archive which exists for the island. However, these results agreed with the observations made by [14, 30] of a higher coherence in the L-band for non-urban areas. Moreover, a high coherence level has also been obtained with images acquired during the unfavourable wet season. The L-band is, therefore, more adapted to the regional context than the C-band.

**Figure 2.**
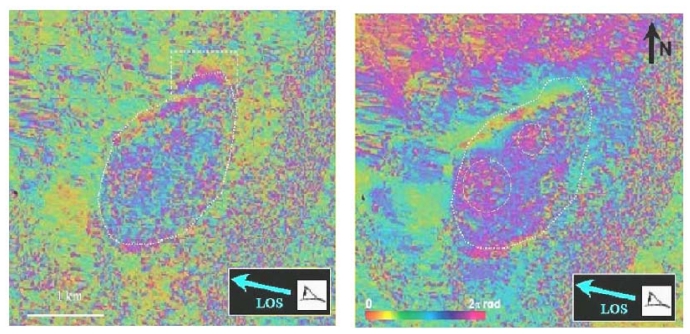
JERS-1 interferogram–left: 02/01/1997-31/03/1997. Right: 15/02/1997-14/05/1997. One color cycle corresponds to 11.25 cm displacement in line of sight (LOS), whose direction is represented by the arrows. White frames give the limits and maxima of the observed displacements.

According to ground observations and GPS measurements, the Hellbourg landslide in the Salazie Circle sector should fall within the limits of application for DInSAR (displacement rates ranging between a few centimetres to a decimetre per month and slopes lower than 15°). We therefore focussed our analysis on this particular area. On the Hellbourg landslide, two independent JERS-1 differential interferograms (02/01/1997-31/03/1997 and 15/02/1997-14/05/1997) clearly exhibit fringes associated to surface motion (Figure 2). This indicates that the observed phase variation is not due to an atmospheric effect which varies randomly form one image to the other but is rather due to ground movement.

**Figure 3.**
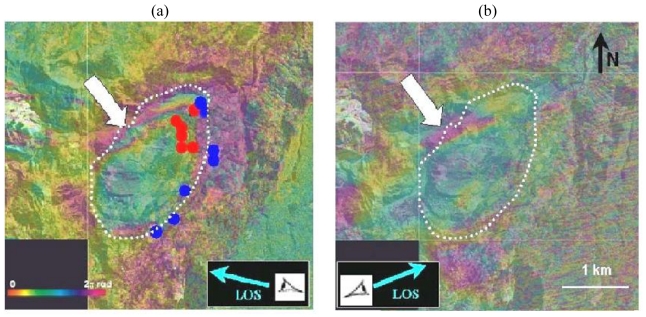
Up: stacking sum of two 3–months L-Band JERS-1 interferograms (1 fringe = 5.6 cm in line of sight). The boundaries (white frames) of the landslide are clearly delimited by the fringe pattern. Circles correspond to GPS points (blue less than 6 cm displacement, red: more than 12 cm displacement). Down: 08/06/2001-26/07/2001 Radarsat C-Band interferogram (1 fringe=2.8 cm in line of sight). The white arrows indicate an unstable area on the left border of the landslide. Despite the poor coherence level in C-band, this interferogram confirms the detection made using the L-band. Background: aerial photograph (source IGN).

The dotted circles on Figure 2 left show areas that seem to move slightly faster (along the LOS) than the rest of the landslide. The white frame in the north east of the landslide points a fringe pattern that is not confirmed on the right image. It could be a displacement which occurred during the period 02/01/1997-15/02/1997. The observed signatures correspond to less than three fringes. An interferogram stacking technique has been applied [33] (Figure 3, left). The deformation signature adds linearly whereas the atmospheric phase seen from independent interferograms adds as their square root [27]. One fringe on this image corresponds to a displacement of 5.6 cm in the line of sight on the satellite.

Only one Radarsat differential C-band interferogram has a non zero coherence level in our study area in the Circles region (08/06/2001-26/07/2001, Figure 3b). All other Radarsat combinations appear to be almost completely noisy for this area. The displacement fringe pattern observed with JERS-1 (Figure 3 left) also appears in the 48 days Radarsat interferogram (Figure 3, right). This confirms our interpretation in terms of ground displacement and rules out any atmospheric effect. One fringe (corresponding to a mean displacement rate of ∼3 cm /month) is detected at the edge of the landslide. This is therefore fully consistent with the L-band measurement.

Ground based GPS measurements have also been acquired in this area. They are reproduced as blue and red dots in Figure 3a.The limits of the landslide detected by DInSAR are fully consistent with the available GPS measurements (coloured circles). However, the DInSAR study provides a better delimitation of the landsliding area in particular in the North-western part where no GPS measurements are available. The upper left part of the landslide (white arrow), which is the most active, shows up to 1.5 fringes. This corresponds to a displacement of 8.4 cm in three months.

**Figure 4.**
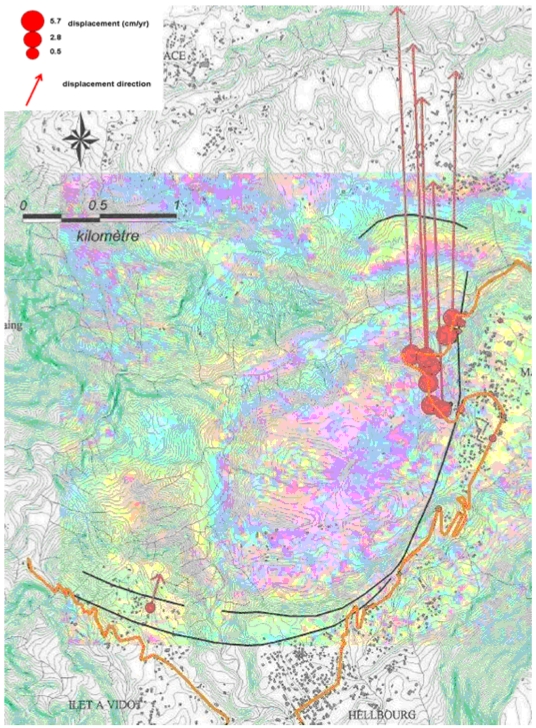
Sum of two 3 months JERS-1 interferograms (1 fringe = 5.6 cm in line of sight) overlain on the topographic map of the landslide (source: Cruchet, 2000). In red, GPS displacements for the period 1995-1999. Black lines represent positions of crack boundaries of the landslide observed by Cruchet. Black-and-yellow line shows the position of the Hellbourg road severely affected by the landslide.

Figure 4 shows the consistency between DInSAR and ground observation (cracks location, GPS measurements). The upper left part of the landslide (white arrow in Figure 3a), which is the most active area, shows up to 1.5 fringe. This corresponds to a displacement of 8.4 cm in 3 months.

## 4. Optical image correlation

The conventional DInSAR method provides a good localization and a first estimation of the displacement in the line of sight of the satellite. For a more comprehensive understanding of the phenomenon, we need an estimation of the three components of the displacement. This information will be obtained by estimating the planimetric displacement field using correlation of multitemporal optical image.

### 4.1. Methodology and data used

To estimate the ground displacement that occurred between two scenes acquired over the same area at two different times, a local window of a fixed number of pixels in width is defined on the oldest orthorectified image. Then, the corresponding window is searched on the more recent orthorectified image by maximizing a correlation function [35]. The starting point of the search is the expected position of the window if no displacement had occurred between the two acquisitions. The measured shift is directly related to the ground displacement between the two acquisitions. The main parameters of the process are the size of the local window and the maximum displacement expected between the images. This process is iterated for each pixel of the oldest image. The result is composed of three arrays that have the size of the correlated images. The first one contains the shift in lines for each pixel, the second contains the shift in columns, and the third indicates the quality of the correlation.

This technique has already been successfully applied to map surface displacement related to earthquakes [36–38], landslides [16, 18], glacier flow [39, 40] and volcanoes [41]. The processing has been carried out using the MEDICIS correlator developed by CNES [42]. This correlator uses FFT for the maximization of correlation ratio. Input parameters are the size of the research window and the size of the sliding windows. The result consists in the tables of x and y displacement and correlation values described above.

For this study two different categories of optical data have been used: an aerial photograph acquired by IGN (Institut Géographique National) in July 1997 with a spatial resolution of 1 m, and a SPOT 5 image (THX band) acquired on 06/07/2002 with a resolution of 2.5 m and a reduced cloud cover on the Salazie Circle area. The SPOT 5 image has been selected in order to limit sun shadows effects (both aerial and SPOT5 images have been acquired July). Furthermore in order to reduce topographic effect acquisitions have to be close to the nadir. No atmospheric compensation has been applied. The two images have been orthorectified and projected in the Gauss Laborde projection. The aerial photograph was chosen as a reference and has been undersampled for a 2.5 m pixel size. For details on the processing, the reader can refer to [40]

### 4.2. Results

**Figure 5.**
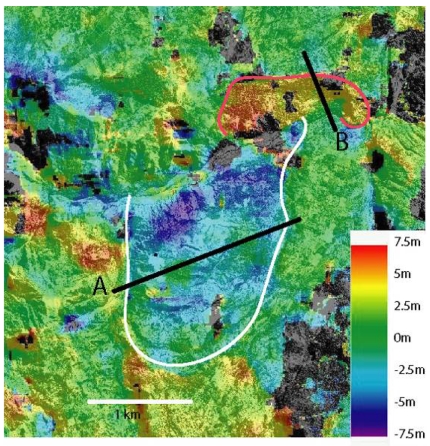
Displacement map along the north-south direction for the Hellbourg area during the 1997-2002 period. The red contour corresponds to a landslide located north of the river, sliding in the southern direction. The white contour corresponds to the boundaries of the main landslide, moving northward. A and B are the profiles presented in Figures 6 and 7. Black areas correspond to low correlation value.

Figure 5 shows the line shifts (north-south direction) between the two images. Size of the local correlation window is 16 × 16 pixels (40 m × 40 m). Shifts along columns appear to be hampered by noise. This noise comes from the acquisition geometry of the SPOT scenes, which is more sensitive to topography in the direction perpendicular to the satellite orbit. Quantification and correction of this noise will be carried out in further studies. The topography effect is less important for the line correlation component.

Two areas with significant displacement can be observed. One is located in the northern part of the image (red contours). Displacements indicate a motion toward the south, which is in agreement with the local slope. The average displacement is around 5 m over five years and the maximum displacement is 7.5 m. The second landslide is located in the southern part of the image and corresponds to the map of the Hellbourg landslide (white contours).

**Figure 6.**
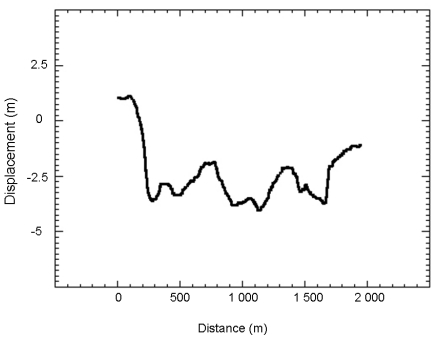
Displacement profile A in Figure 4, along the North-South direction during the 1997-2002 period. Horizontal axis corresponds to distance (in m) along the profile.

Some other patterns can be observed outside the landslides area (black circles in Figure 6). They are not associated to motions. They can be explained by surface state changes, shadows, topographic effects coming from orthorectified errors [16, 40].

**Figure 7.**
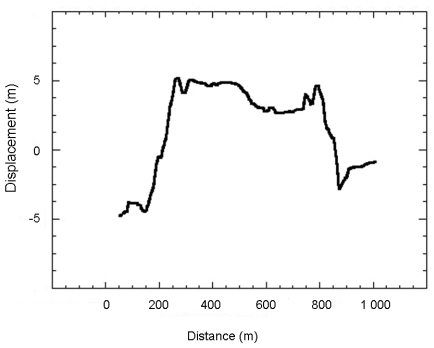
Displacement profile B in Figure 5, along the North-South direction during the 1997-2002 period. Horizontal axis corresponds to distance (in m) along the profile.

To assess the rate of displacement that occurred during the period between the two acquisition dates, two profiles (A and B in Figure 5) have been extracted (Figures 6 and 7). Irrelevant points have been rejected by applying a 10 m threshold to the obtained values. Spatial heterogeneities of the displacement are clearly visible.

In five years, the planimetric displacement of the southern landslide, measured perpendicularly to the movement direction, is between 2 and 5 meters. On the Figure 6, the value of displacement is negative because the displacement is directed toward the North. The displacement is not completely homogeneous. On spatial wavelengths of around 500 m, the displacement oscillates by about 1 meter. Two clear low velocity patches, visible in the upper part of the landslide (Figure 5) are crossed by the profile. The displacement of these patches is close to the displacement measured on the stable areas and thus could be only noise. These patches could be stable. The eastern patch corresponds to a small lake, on which correlation is impossible. The western one corresponds to a vegetated East-West elongated hill, for which the correlation process is more difficult. Thus, the low value of displacement could be related only to difficulties in correlation. Displacement values averaged over the three months are of 25 cm. In five years, the planimetric displacement of the northern landslide is of 6 meters when measured in a North-South direction and along the steepest slope. This landslide is smaller than the southern one and the displacement is thus more homogeneous. The values of displacement averaged on three months are of 50 cm. This landslide is clearly more active than the southern one.

## 5. Discussion and Conclusions

Among the two remote sensing techniques used to characterize the Hellbourg landslide on the island of la Reunion in the Indian Ocean, theDInSAR analysis, carried out with 14 Radarsat C-band images and a set of six JERS-1 L-band images, shows the interest of L-band DInSAR. Both the shape and the Line Of Sight displacement rate of the Hellbourg landslide have been estimated and are compatible with groundbased GPS measurements. Displacements larger than one fringe (i.e., about 11.5 cm) were observed. These results show the interest of using L-band data in a context where the C-band is not appropriate, due to the vegetation cover. The main drawback of the C-band comes from the sensitivity of the radar signal to the vegetal cover, which induces a strong coherence loss in the interferograms. In a highly vegetated area such as the La Réunion Circles, this is a very limiting factor which precludes the use of large time spans.

The reduced JERS-1available archive (only six images in 1997) precludes a comprehensive analysis of the landslide's kinematic evolution. Our results show that L-band missions (such as ALOS/PALSAR for producing a sufficient archive for this area) could play a significant role in the very near future for the monitoring of landslides in vegetated areas. Provided that a homogeneous archive is progressively built, such a remote sensing technique could provide an operational tool that is complementary to ground-based techniques for the mitigation of landslide risk. We have used the correlation of optical data to derive the 2D horizontal motions of the landslide over several years. The correlation between SPOT 5 image acquired in 2002 and aerial optical images acquired in 1997, have permitted the detection of displacements between 5 and 7.5 m over a period of five years. Both the geometry and kinematic evolution of the two landslides studied in the Hellbourg area by optical correlation are compatible with the DInSAR results. Because of different time spanned between DInSAR and correlation, it is difficult to precisely compare the displacement values obtained by the two techniques. Furthermore, DInSAR gives a 1D component of the displacement (along the line of sight) and optical correlation gives 2D component (in the horizontal plan). So full 3D motion of the landslide cannot be derived directly from the displacements maps without strong assumptions on the other displacement components. This technique can be applied either with two scenes acquired at two periods by the same type of sensor, or with different sensors (for example aerial photography combined with very high resolution satellite imagery, as was achieved here). This technique allows the mapping of a large displacement field. The complementarity of DInSAR and optical correlation provides 3D information on the displacements. DInSAR gives the displacement along the line of sight (with an important vertical component for most satellites) with a centimetric accuracy and optical correlation gives 2D planimetric motion with decimetric accuracy (1/10 of spatial resolution according to [17]). Indeed, optical correlation can be applied over few years whereas in vegetated area the loss of coherence prevents the use of DInSAR with images acquired few years apart. Furthermore, the spatial accuracy of optical correlation is higher. Indeed, aerial or very high resolution images provide submetric spatial resolution. This spatial resolution allows the better discrimination of spatial heterogeneities of the motions. The increase in spatial resolution of the new satellites (IKONOS, Quickbird, ORFEO) will allow a better accuracy in the computation of lateral displacements using correlation techniques once a comprehensive archive will be acquired. Thanks to those new data, we can expect that combination of DInSAR and optical correlation will be widely used for landslides studies.

## Figures and Tables

**Table 1.** Characteristics of the eleven most coherent RADARSAT inteferograms used for the interferometric study. Perpendicular baselines of the interferometric pairs (in meters) are indicated in the lower part of the table. The baseline is the distance between the two satellites when the images have been acquired. Long baselines produce loss of coherency in the interferograms. The upper part gives the time spans in days.

Date	Orbit	16850	18908	19594	21652	21995	22338	23710	24053	25768	26797	28855	29198	29884	31599	32285
	Orbit															
26/01/1999	16850										225					
13/07/1999	18908															
06/08/1999	19594					85						235				
28/12/1999	21652						190									
21/01/2000	21995			168									115	82		
14/02/2000	22338				48											
20/05/2000	23710															
13/06/2000	24053										360		260			
11/10/2000	25768															280
22/12/2000	26797	696							192							
15/05/2001	28855			648											68	
08/06/2001	29198					480			360					35		
26/07/2001	29884					528							48			
23/11/2001	31599											192				
10/01/2002	32285									440						

**Table 2.** JERS-1 data used for the interferometric study. Perpendicular baselines of the interferometric pairs (in metres) are indicated in the lower part of the table. The upper part gives the time spans in days.

Date	02/01/1997	15/02/1997	31/03/1997	14/05/1997	27/06/1997	10/08/1997
02/01/1997		44	88	132		
15/02/1997	153		44	88		
31/03/1997	111	70		44		
14/05/1997	187	99	90			
27/06/1997						44
10/08/1997					920	
